# Clinicalpathologic and Prognostic Significance of CGI-58 in Endometrial Cancer

**DOI:** 10.7150/jca.61905

**Published:** 2021-10-28

**Authors:** Zhengzheng Shi, Xishao Luo, Hongqin Zhao, Baoyou Huang, Yuanqiu Wang, Xu Chen, Jiangtao Yu

**Affiliations:** Department of Gynecology, the First Affiliated Hospital of Wenzhou Medical University, Wenzhou 325027, China.

**Keywords:** CGI-58, Endometrial cancer, Immunohistochemistry, Prognosis

## Abstract

Recent studies have reported that CGI-58 played an important role in carcinogenesis and tumoral progression in several cancers. In this study, we investigated the expression and prognostic value of CGI-58 in patients with endometrail cancer. Initially, the expression of CGI-58 was analyzed in 552 cases of endometrial carcinoma from The Cancer Genome Atlas (TCGA). Then, the mRNA level of CGI-58 from 32 normal endometrium and 40 endometrial cancer tissues was determined using real-time PCR. In addition, immunohistochemical staining of CGI-58 was performed in 140 endometrial specimens including 35 normal endometrial tissues, 25 atypical endometrial hyperplasia and 80 endometrial cancers. The expression of CGI-58 was significantly up-regulated in endometrial cancer tissues compared with normal endometrial tissue both in TCGA database and clinical cohorts. Over-expression of CGI-58 was significantly correlated with poor histological differentiation. Furthermore, high levels of CGI-58 expression were significantly associated with shorter overall survival for all analyzed cases. Our findings demonstrate that CGI-58 is up-regulated in endometrial cancer and high CGI-58 expression is a poor prognostic marker for endometrial cancer. CGI-58 may be a potential contributor to endometrial cancer oncogenesis and progression.

## Introduction

Endometrial cancer was the second most commonly diagnosed gynecological cancer worldwide, with an estimated 65,620 new cases in 2020 1. Although hysterectomy was curative in most patients with well-differentiation and early stage diseases, the outcomes for poor-differentiation and metastatic/recurrent cases remained poor 2. Therefore, it is of great clinical value to discovery and characterization of new biomarkers to evaluate more accurately the prognosis and thus to assess the treatment options of individual patients.

Comparative gene identification 5 (CGI-58), also known as 58α β hydrolase domain-containing (ABHD5), was a lipolytic co-activator that promotes the hydrolysis of triglyceride by activating adipose triglyceride lipase 3, 4. Mutation of CGI-58 was well known as a cause for Chanarin-Dorfman syndrome, a condition in which triglyceride accumulates in multiple organs or tissues 3, 4. Recent studies had indicated that CGI-58 played an important role in carcinogenesis and tumor progression in several cancers, such as colorectal cancer 5 and prostate cancer 6. CGI-58 was over-expressed in colorectal cancer-associated macrophages and functioned as an oncogene 5. Mitra et al. reported that CGI-58 was up-regulated in prostate cancer cells compared to peripheral blood mononuclear cells 6. CGI-58 inhibition by siRNA suppressed the growth of prostate cancer via triggering apoptosis 6. However, the roles of CGI-58 in endometrial cancer have not been elucidated.

In the study, we explored the expression of CGI-58 and its prognostic role in human endometrial cancer using TCGA database, and then confirmed these results by immunohistochemistry and real-time PCR in two endometrial cancer cohorts, respectively.

## Materials and Methods

### Ethics statement

This study was approved by the ethical committee of the First Affiliated Hospital of Wenzhou Medical University and conducted according to the Helsinki declaration. A written informed consent was granted from all subjects at the time of enrollment.

### TCGA

Briefly, RNA sequencing data (552 uterine corpus endometrial cancer and 23 normal endometrial samples) were downloaded from TCGA database (https://gdc-portal.nci.nih.gov/), including clinical data and CGI-58 mRNA level.

### Patients and tissue specimens

Two groups of human endometrial cancer specimens were included in the current study, Cohort 1 included 32 normal endometrial tissues and 40 endometrial cancer tissues, which were collected from patients at the First Affiliated Hospital of Wenzhou Medical University between January 2018 and October 2018. In cohort 2, a total of 35 normal endometrium, 25 atypical hyperplasia endometrium, and 80 endometrial cancer tissues were collected from patients who underwent surgical resection at the First Affiliated Hospital of Wenzhou Medical University, Wenzhou, China, between January 2010 and December 2013. We had carefully screened about the patients and none of the patients included in the study had serious medical and surgical diseases, such as hypertension or diabetes, etc. All patients were aged 18 years or older and had adequate function of major organs (e.g. heart, liver, kidneys). Eligible patients had histologically confirmed and all tissue samples were obtained from patients without undergoing any medical treatment before surgery. Exclusion criteria were uterine sarcoma or cancer in other systems. Patients were also excluded if they had previous pelvic radiotherapy, hormonal therapy, chemotherapy, or impaired renal or cardiac function.

### Follow-up data

Detailed clinicopathological information and follow-up record were collected from all 80 cases of endometrial cancer patients in cohort 2. Patients in cohort 2 were followed up in a post-operative outpatient schedule every 3 months for 2 years, every 6 months thereafter for a total of 3 years and every 1 year thereafter. The deadline of follow-up time was Dec 1, 2018. Overall survival (OS), the primary end point of our study, was calculated from the date of surgery to the date of death.

### Real-time PCR

RNA extracted from the frozen human tissues was treated with DNase1 (Invitrogen) to remove genomic DNA contamination. Total RNA (2 mg) was reverse-transcribed into cDNA using random primers and M-MLV reverse transcriptase from Invitrogen Life Technology. Quantitative real-time PCR (qRT-PCR) was performed using Power SYBR Green PCR Mix from Life Technologies. All primers used in this study were designed by Primer Blast of NCBI and synthesized by Integrated DNA Technologies. Primer pair specificity was determined by generation of a single peak for dissociation curve (melting curve) at the end of real-time PCR cycling program. GAPDH was used as the internal control.

### Immunohistochemistry

Immunohistochemical specimens of endometrial carcinoma and endometrial atypical hyperplasia came from the specimens after hysterectomy, while normal endometrium came from diagnostic curettage or hysterectomy. Specimens were embedded in paraffin, cut into 4 µm sections, and then mounted onto poly-L-lysine-coated slides. In briefly, after deparaffinization in xylenes and rehydration through graded ethanol solutions, sections were boiled in a 10 µmol/L citric buffer solution (pH 6.0) in a microwave oven for 10 minutes, followed by incubation with 3% hydrogen peroxide in methanol to suppress the endogenous peroxidase activity and overnight incubation with primary antibodies at 4 °C. The following primary antibodies and corresponding dilutions were used: CGI-58 (Abcam, USA, 1:200). Subsequently, the sections were incubated with pre-diluted biotinylated secondary antibody for 2 hours at room temperature, followed by further incubation with 3, 3-diaminobenzidine tetrahydrochloride (DAB). Finally, the slides were counterstained with hematoxylin and mounted in an aqueous mounting medium. Appropriate positive and negative controls were stained in parallel. For negative controls, primary antibodies were replaced with Phosphate-buffered Saline Solution (PBS). Human prostate cancer tissues were used as a positive control.

### Immunohistochemical staining analysis

Staining evaluation was assessed by two independent observers, who were blinded to the clinical outcome. With the help of computer image analysis system, we used the method of average positive stained area to evaluate the slice immunohistochemistry results 7. Briefly, Olympus BX43, image acquisition software microview snap300, and image analysis software were used. For each case, acquisition was performed in 5 representative regions with Olympus BX43 at ×100 magnification. Then, all the photographs were analyzed by image analysis software. Finally, positive staining area was calculated.

### Statistical analysis

Continuous data with a normal distribution were expressed as the mean ± standard deviation (SD) and compared using a standard *t* test. Continuous data with non-normal distribution were presented as median ± interquartile range (IQR) and compared using the Wilcoxon rank-sum test. The frequencies of categorical variables were compared using Chi-square or Fisher's exact test, when appropriate. The survival analyses were explored through the Kaplan-Meier method with a log-rank test. The significance of the variables for survival was analyzed by univariate and multivariate Cox regression analysis. The software of GraphPad Prism and SPSS were used for statistical analysis. A *P*-value less than 0.05 were considered statistically significant.

## Results

### CGI-58 is up-regulated in endometrial cancer and predicts a poor OS in the TCGA database

**Figure 1:**
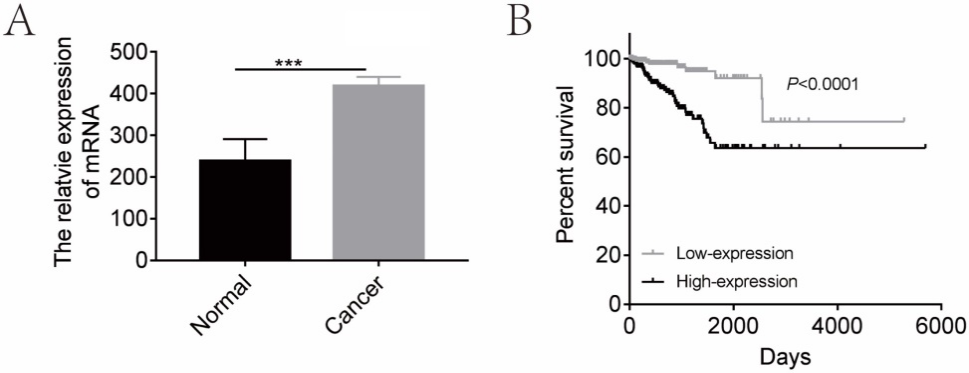
** CGI-58 is up-regulated in human endometrial cancer tissues and predicts a poor prognosis using TCGA database.** (A) The expression of CGI-58 is up-regulated in endometrial cancer as compared with normal endometrial tissues. (B) High-expression of CGI-58 is associated with shorter OS of endometrial cancer patients. *P < 0.05, **P < 0.01, ***P < 0.001.

In order to determine the clinical relevance of CGI-58 expression, we first analyzed mRNA levels of CGI-58 in 552 human endometrial cancer and 23 normal endometrium tissues using TCGA database. As shown in Figure 1, mRNA levels of CGI-58 were significantly higher in cancer tissues than that in normal endometrial tissues. We further explored the relationship between CGI-58 mRNA levels and clinicopathological features among patients with endometrial cancer. The expression cut-off point was determined according to median CGI-58 mRNA levels, thus, CGI-58 expression status was classified into “low” and “high”. As shown in Table 1, high CGI-58 mRNA level was significantly correlated with tumor grade and histology, implying other specific types of endometrial cancer and poor differentiation were often associated with up-expression of CGI-58, but not with age, FIGO stage (Table 1).

We then evaluated the prognostic effects of high CGI-58 expression in endometrial cancer. Using the log-rank test (Figure 1), high-expression of CGI-58 was associated with a significantly worse OS (high-expression vs low-expression, HR= 4.363, 95%CI: 2.506 -7.596, *P* < 0.0001).

### CGI-58 is over-expressed in human endometrial cancer samples

**Figure 2:**
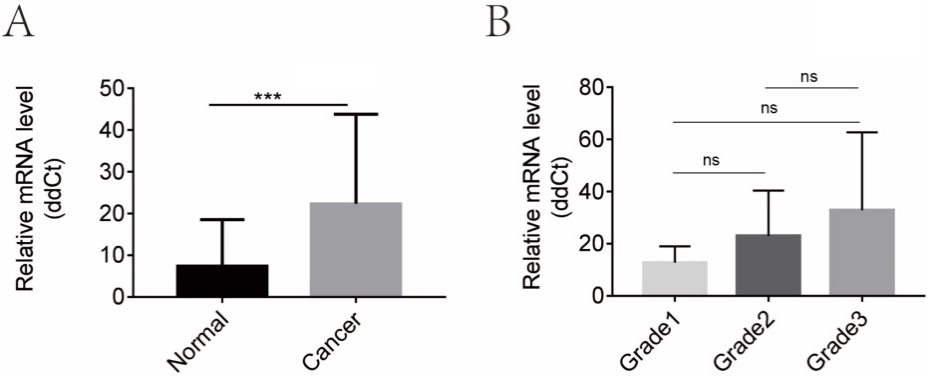
** CGI-58 is up-regulated in human endometrial cancer tissues and increased with the histopathologic grade in clinical cohort 1.** (A) The mRNA expression of CGI-58 is up-regulated in endometrial cancer as compared with normal endometrial tissues. (B) The mRNA level of CGI-58 increased with the histopathologic grade of endometrial tissues (Grade 1 < Grade 2 < Grade 3). *P < 0.05, **P < 0.01, ***P < 0.001. Abbreviations: ns = Not Statistically Significant.

We then evaluated the mRNA levels of CGI-58 in cohort 1 using real-time PCR, including 32 cases of normal endometrial tissues and 40 cases of endometrial cancer tissues. As shown in Figure 2, the mRNA expression of CGI-58 was significantly higher in endometrial carcinoma than that in normal endometrium (*P* < 0.001). In addition, the mRNA level of CGI-58 increased with the histopathologic grade of endometrial tissues (Grade 1 < Grade 2 < Grade 3), with a* P* value > 0.05.

**Figure 3:**
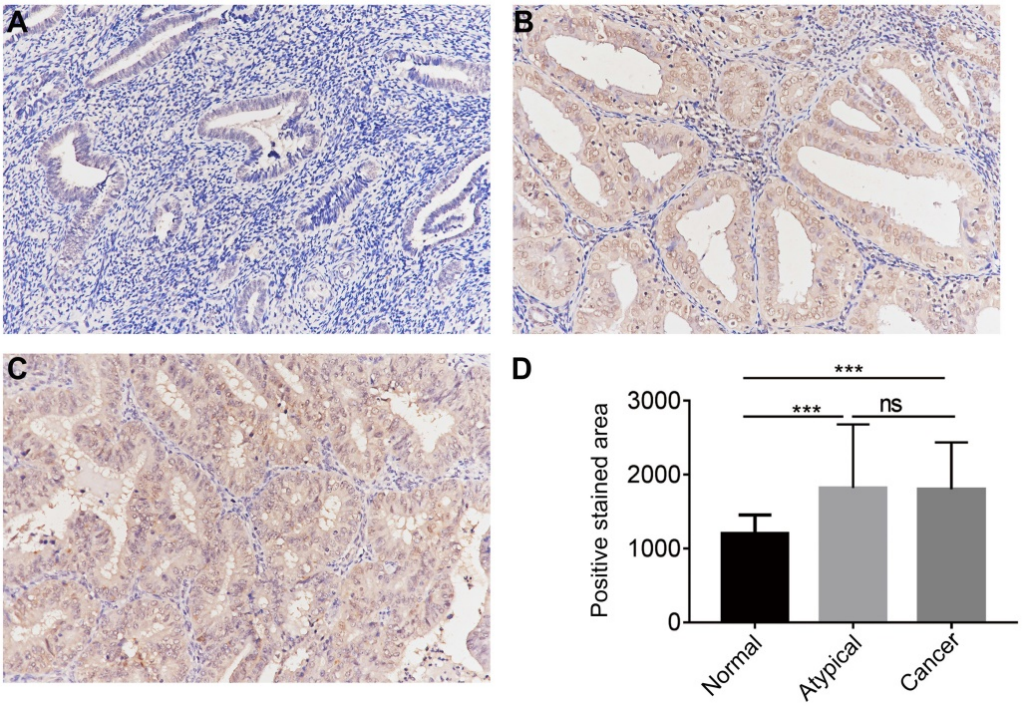
** The protein expression of CGI-58 in endometrial cancer and normal endometrial tissue (SP staining, × 200) in clinical cohort 2.** Immunohistochemical staining slides showed weak positive CGI-58 staining in normal endometrium (A), and moderate or intense positive stain in atypical endometrial hyperplasia (B) and endometrial cancer (C). Compared to normal endometrial tissue, CGI-58 showed significantly elevated positive staining area in atypical endometrial hyperplasia and endometrial cancer. *P < 0.05, **P < 0.01, ***P < 0.001. Abbreviations: ns = Not Statistically Significant.

The protein expression of CGI-58 in cohort 2, including 35 normal endometrial tissues, 25 atypical endometrial hyperplasia and 80 endometrial cancers samples, was then evaluated using immunohistochemistry. As shown in Figure 3, CGI-58 protein primarily localized in the cytoplasm. Endometrial cancer and atypical endometrial hyperplasia tissues consistently showed moderate or intense positive stain (Figure 3B and C), while normal tissues consistently showed negative or weak positive staining (Figure 3A). The expression of CGI-58 was significantly increased in cancer samples and atypical endometrial hyperplasia compared with normal tissue samples, (*P* < 0.001, respectively) (Figure 3D). Interestingly, there showed no significant difference between atypical endometrial hyperplasia and endometrial cancer (Figure 3D).

We further explored the correlation between protein expression of CGI-58 and clinicopathological characteristics. As shown in Table 1, high-expression of CGI-58 was associated with grade, but not with age, FIGO stage, and histology.

### CGI-58 is associated with a poor OS in human endometrial cancer samples

**Figure 4:**
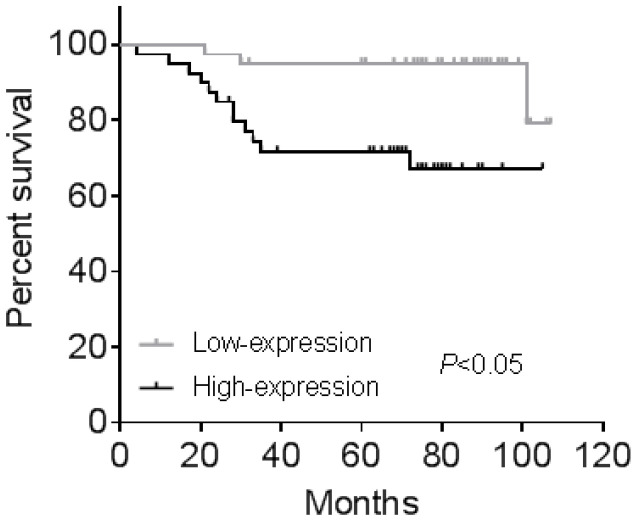
** Kaplan-Meier survival curve for endometrial cancer patients stratified by CGI-58 expression in clinical cohort 2.** Human endometrial cancer patients with high-expression of CGI-58 had shorter OS comparing to patients with low-expression of CGI-58.

All the 80 cases of endometrial cancer patients from cohort 2 were divided into high-expression and low-expression groups according to median value of IHC positive stained area. Kaplan-Meier analysis showed human endometrial cancer patients with high CGI-58 expression had shorter OS (*P* < 0.05, Figure 4) than that with low- expression of CGI-58. In addition, using univariate Cox model, FIGO stage, grade and CGI-58 expression were significantly correlated to OS in cohort 2 (Table 2). Multivariate analysis performed using the Cox proportional hazards model indicated that CGI-58 expression was an independent poor prognostic factor for OS in patients with endometrial cancer, with a HR of 4.054 (95% CI: 1.009~16.280, *P* = 0.048).

## Discussion

In the present study, we report that CGI-58 is up-regulated and predicts a worse clinical outcome in human with endometrial cancer both in TCGA cohort and two clinical cohorts, suggesting that CGI-58 may be involved in the oncogenesis and progression of endometrial cancer. To our knowledge, this is the first study to investigate the role of CGI-58 in human endometrial cancer.

CGI-58 is well known as a lipolytic co-activator, which involved in lipolysis of triglycerides into diglycerides and free fatty acids 3, 8. Recently, some studies showed that CGI-58 might influence cancer metabolism, thus, involved in cancer initiation and progression 9. Senchenko et al. detected high frequencies of methylation/deletion of CGI-58 during cervical carcinogenesis, using original technology of NotI-microarrays 10. Ou et al. reported CGI-58 was downregulated and functioned as a tumor suppressor in colorectal cancer in 2014 11. Using a combination of genetically engineered mice model and molecular cellular approaches, they found CGI-58 deficiency promoted colorectal tumor development by inducing glycolysis and epithelialmesenchymal transition 11. Interestingly, in 2016, the same researcher reported that CGI-58 expression was increased in tumor-associated macrophages and the increased CGI-58 in macrophages facilitated the growth of colorectal cancer cells by suppressing spermidine synthase-dependent spermidine production in macrophages 5. Mitra et al. 6 reported that CGI-58 was over-expressed in prostate cancer cells and inhibition of CGI-58 lead to cell cycle block and cell death by triggering apoptosis. Whereas Chen et al. 12 reported markedly decreased CGI-58 gene expression in metastatic castration-resistant prostate cancer samples using publicly accessible prostate cancer gene expression datasets and RNAi-mediated CGI-58 silencing promoted the invasion and proliferation of prostate cancer cell by inducing epithelial to mesenchymal transition and the warburg effect. The seemingly opposing studies indicate a tissue/cell-specific role of CGI-58. In this study, we used several detection approaches and confirmed our results in TCGA database as well as in two clinical cohorts. We found that the expression of CGI-58 was up-regulated in endometrial cancer both in mRNA and protein level, suggesting CGI-58 involved in endometrial carcinogenesis.

We further analyzed the correlation between the expression of CGI-58 and clinical characteristics, and found high-expression of CGI-58 in endometrail cancer was significantly correlated with poor differentiation both in TCGA database and clinical cohorts. Tumor grading is well demonstrated as a poor prognostic factor in patients with endometrioid carcinomas 13. Consistence with previous reports, our results showed poor grade was associated with short OS in endometrial cancer patients. These results further supported the notion that CGI-58 might function as a contributor in endometrial cancer. In our previous research 14, some data implied that ABHD5 might affect endometrial cancer cell invasion via regulating EMT transition. In ABHD5 knockdown cells, which further confirmed the reduction in the Warburg effect. In the future study, we will focus on the mechanism of CGI-58 in endometrial cancer. This is the direction of our research based on *in vivo* and *in vitro* experiments.

To the best of our knowledge, this is the first study to evaluate the prognostic role of CGI-58 in endometrial cancer. Our current results demonstrated that high-expression of CGI-58 are associated with a poor OS in human endometrial carcinoma using univariate as well as multivariate Cox regression analysis. In all, CGI-58 may be a potential marker of poor prognosis in human endometrial cancer. Further study with more samples is needed to demonstrate whether it is an independent prognostic factor.

In conclusion, our current study demonstrates an increased expression of CGI-58 in endometrial cancer. It is also suggested from our findings that high expression of CGI-58 predicts a worse prognosis in endometrial cancer. Our future study will further explore the role of CGI-58 in endometrial carcinogenesis* in vitro* and* in vivo*.

## Figures and Tables

**Table 1:** The relationship between CGI-58 and clinicopathologic characteristics in patients with endometrial cancer.

Characteristics	TCGA				Cohort 2			
	n	Low (%)	High (%)	*P*	n	Low (%)	High (%)	*P*
Age				0.605	80			0.152
<=60	206	101 (49)	105 (51)		54	30 (55.6)	24 (44.4)	
>60	343	176 (51)	167 (49)		26	10 (38.5)	16 (61.5)	
Missing information	3							
Histology				0.025*				0.176
Endometrioid-type	410	195 (47.6)	215 (52.4)		70			
serous carcinoma	142	83 (58.5)	59 (41.5)		10	37 (52.9)	33 (47.1)	
						3 (30)	7 (70)	
FIGO Stage				0.055				0.210
I-II	393	187 (47.6)	206 (52.4)		68	36 (52.9)	32 (47.1)	
III-IV	159	90 (56.6)	69 (43.4)		12	4 (33.3)	8 (66.7)	
Differentiation				<0.001*				0.004*
1	109	74 (67.9)	35 (32.1)		32	23 (71.9)	9 (28.1)	
2	120	65 (54.2)	55 (45.8)		26	11 (42.3)	15 (57.7)	
3	323	138 (42.7)	185 (57.3)		22	6 (27.3)	16 (72.7)	
Death				0.092				0.01*
No	507	249 (49.1)	258 (50.9)		65	37 (56.9)	28 (43.1)	
Yes	45	28 (62.2)	17 (37.8)		15	3 (20)	12 (80)	

Abbreviations: n = number of patients;

**Table 2:** Univariate and Multivariate Cox regression analysis for overall survival of patients with endometrial cancer

Variates	Univariate		Multivariate	
	HR(95% CI)	*P*	HR(95% CI)	*P*
Age				
<=60 vs >60	2.119(0.763-5.882)	0.150	1.994(0.547-7.272)	0.296
Histology				
Endometrioid vs Other-type	2.078(0.577-7.483)	0.263	0.910(0.202-4.094)	0.902
FIGO Stage				
I-II vs III-IV	8.006(2.791-22.963)	0.000*	6.975(2.207-22.041)	0.001*
Differentiation				
1-2 vs 3	3.036(1.064-8.665)	0.038*	1.271(0.375-4.308)	0.700
CGI-58				
High vs Low	5.642(1.542-20.648)	0.009*	4.054(1.009-16.280)	0.048*

*: *P<*0.05
